# Influence of customized therapy for molar incisor hypomineralization on children's oral hygiene and quality of life

**DOI:** 10.1002/cre2.245

**Published:** 2019-09-13

**Authors:** Jana Fütterer, Markus Ebel, Katrin Bekes, Christian Klode, Christian Hirsch

**Affiliations:** ^1^ Leo Löwenzahn Pediatric Dentistry Practice Bergisch Gladbach Germany; ^2^ School of Dentistry, Department of Pediatric Dentistry Medical University of Vienna Vienna Austria; ^3^ Department of Business Analytics and Data Science HMKW University of Applied Science Cologne Germany; ^4^ Faculty of Economics and Management, Department of Knowledge Management University of Marburg Marburg Germany; ^5^ School of Dentistry, Department of Pediatric Dentistry University of Leipzig Leipzig Germany

**Keywords:** hypersensitivity, MIH, MIH treatment, MIH Treatment Need Index, nutritional restrictions, oral hygiene

## Abstract

**Objective:**

The aim of this clinical follow‐up study was to demonstrate the effects of different therapeutic strategies for hypomineralized teeth on patients' oral health. The treatment results were characterized by changes in the extent of hypersensitivity and plaque accumulation, as well as reductions in nutritional restrictions.

**Material and Methods:**

The impacts of therapy, including the use of fluoride varnish, fissure sealants, fillings, and stainless steel crowns, were evaluated in 78 children (mean age 8.5 years). We followed recommendations according to the Molar Incisor Hypomineralisation Treatment Need Index for customized treatment. The Quigley Hein Index, the Schiff Cold Air Sensitivity Scale, Wong–Baker Faces Scale, and dietary‐limiting parameters were assessed before and after therapy for comparison.

**Results:**

Plaque accumulation and hypersensitivity decreased after completion of therapy. The improvements were greater for individual teeth (Quigley Hein Index for teeth treated with stainless steel crowns from 4.19 to 2.54) than for those of the whole dentition (high‐severity category from 2.67 to 2.20). Problems with food intake were minimized via therapy, with the greatest influence observed for patients who were also in the high‐severity category.

**Conclusions:**

Therapy for affected teeth in children has positive effects on oral health and quality of life.

## 1 INTRODUCTION

Enamel disorders of molar incisor hypomineralization (MIH) and deciduous molar hypomineralization (DMH) have recently received increasing attention from pediatric dentists worldwide. MIH describes a developmental enamel defect of at least one permanent first molar with or without affected permanent incisors (Weerheijm, Jalevik, & Alaluusua, 2001). Since 2017, it has been shown that MIH/DMH can affect all deciduous and permanent teeth (Baroni & Marchionni, 2010). A recent meta‐analysis of studies determined an average global prevalence of MIH of 14.2% (Dave & Taylor, 2018). Posteruptive enamel fractures can occur in affected teeth, which are frequently associated with hypersensitivity and can lead to poorer dental hygiene, problems with the intake of food (PwIoF), and decreased oral health‐related quality of life (Ebel et al., 2018). According to the fifth German Oral Health Study (DMS V), the occurrence of MIH/DMH in 12‐year‐old children in Germany is higher than that of caries among this population (Bekes & Hirsch, 2013). It has been shown that eight out of ten 12‐year‐old children are free of tooth decay (81.3%), but the same group has a 28.7% (Lygidakis et al., 2010; Weerheijm et al., 2003) prevalence of MIH. This trend emphasizes the extraordinary importance and attention among pediatric dentistry worldwide and explains the variety of research in this field.

Ebel et al. (2018) verified severity‐related consequences for the oral hygiene and quality of life of MIH‐/DMH‐affected children. The quantity and extent of structural disorders of affected teeth were correlated with increased consequences and restrictions: with the increasing destruction of teeth accompanied by increased hypersensitivity, as determined by the Schiff Cold Air Sensitivity Scale (SCASS) and Wong–Baker Faces Scale (WBFS), plaque accumulation (Quigley Hein Index [QHI]) significantly increased. In addition, the risk of dietary restrictions, characterized by pain during eating and drinking, as well as pain during domestic and professional dental care, increased with the disease severity and had a negative impact on the children's development and susceptibility to caries (Jälevik & Klingberg, 2002; Petrou et al., 2014).

Although studies and scientific research on MIH/DMH promote awareness among pediatric dentists, no standardized treatment currently exists. A major issue challenging the assortment of optimal treatment modalities is the diversity of the disorder, with its different severity levels, including its various side effects, and the broad spectrum of available therapeutic approaches.

The MIH Treatment Need Index (MIH‐TNI), introduced by Steffen et al. (2017), presents recommendations for decision making when planning individual treatment of MIH‐affected teeth (Jälevik, 2010; Steffen et al., 2017). The MIH‐TNI differentiates affected teeth according to the presence of hypersensitivity and the extent of enamel defects. Furthermore, a treatment scheme has been developed for patients with a low or high risk of developing caries. The TNI's therapeutic recommendations include pure dental prophylaxis such as with fluoride varnish and fissure sealant and also restorative restorations such as fillings, crowns, and extraction.

The purpose of this study was to investigate the influence of customized therapeutic approaches based on the MIH‐TNI on the disease‐associated parameters investigated by Ebel et al. (2018). The study sought to determine whether treatment of the affected teeth could lead to improved oral hygiene (QHI) as well as the extent of hypersensitivity (SCASS and WBFS) and nutritional restrictions (PwIoF) that could be reduced as a result of therapy.

## 2 MATERIALS AND METHODS

### 2.1 Study design and ethics

In this follow‐up study with repeated measures, the values for different therapeutic approaches (fluoride varnish, fissure sealant, filling with composite, stainless steel crown, and extraction) were analyzed according to their impact on oral hygiene, hypersensitivity, and oral health‐related quality‐of‐life impairment regarding the severity of MIH/DMH in affected children. The Ethics Committee of the University of Leipzig, Germany (AZ: 187/17‐ek), authorized this project. The patients' legal representatives were informed in oral and written form, and they consented to participate in this study according to the Declaration of Helsinki guidelines.

### 2.2 Setting

This investigation was based on a sample of consecutive patients experiencing hypomineralized teeth, who were recruited from the Leo Löwenzahn pediatric dentistry practice in Bergisch Gladbach, Germany, between January and December 2017. The children were diagnosed on the basis of clinical examination under artificial light and within the same dental unit as described in Ebel et al. (2018). Following an initial examination period of patients from January to December 2017 conducted by the lead dentists (J. F. and M. E.), the patients were treated with MIH severity‐dependent therapy based on the criteria of the MIH‐TNI (Jälevik, 2010; Steffen et al., 2017). Therapy was implemented for an average of 3 months after the initial examination, ranging from 2 to 401 days. Patients were followed up after therapy by JF, on average, after 3 months (varying from 7 to 488 days) in August 2017 to November 2018. The same parameters as in the initial examination were collected.

### 2.3 Participants

In total, 78 consecutive patients suffering from at least one hypomineralized tooth were selected among the patient pool. The guidelines of the European Academy of Pediatric Dentistry (Lygidakis et al., 2010; Schwendicke et al., 2018; Weerheijm, 2003; Weerheijm et al., 2003) were applied to diagnose the MIH‐positive teeth: (a) clearly definable opacity and/or buccal surfaces of the crowns, (b) white‐/yellow‐to‐brown discolorations, (c) MIH defects of at least 1 mm in diameter, (d) the presence of hypersensitivity, (e) the presence of atypical restorations, and (f) the extraction of permanent teeth due to MIH. Patients who did not collaborate willingly were excluded from the survey. In addition, all patients undergoing orthodontic treatment during the study period were excluded, as orthodontic treatment may have a negative impact on the ability of a patient to perform proper oral hygiene.

### 2.4 Sample size

G*Power Software (http://www.gpower.hhu.de) was used to analyze the sample size. Within this follow‐up study, the difference between several premeasurement and postmeasurement (matched) pairs should be tested according to their statistical significance by applying either analysis of variance (ANOVA) using mean differences or Wilcoxon test using rank differences. We took the standard default thresholds (effect size = 0.5) and used 95% confidence intervals due to the common error probabilities of 5%. For the model applications, G*Power suggested a sample size of *n* = 45 for the ANOVA and *n* = 47 for the Wilcoxon test. Although we did not expect significant differences in gender (see Ebel et al., 2018), we drew a larger sample to establish a nearly equal gender ratio. This follow‐up study included data from 78 patients (37 girls and 41 boys).

### 2.5 Variables

The QHI was defined as the quantitative outcome variable. The SCASS and WBFS were defined as intermediating quantitative variables, which may work as dependent or independent variables (see Ebel et al., 2018). The tooth‐related MIH groups (I–III), patient‐related severity (low‐severity category [LSC], medium‐severity category [MSC], and high‐severity category [HSC]), and treatment groups (fluoride varnish, sealant, filling, SSC, and extraction) served as predictors. In addition, we used additional exogenous variables, including gender, social status groups, and age, and two binary control variables: PwIoF and tooth decay risk (TDR).

### 2.6 Measurements

Oral hygiene was measured in terms of plaque distribution, as several studies support the use of the QHI (Quigley & Hein, 1962) to determine overall dental health and caries risk (Menghini, Steiner, & Imfeld, 2008; Newman, 1986; Walsh, 2006). Caries risk was classified according to the recommendations of the Deutsche Arbeitsgemeinschaft für Jugendzahnpflege e.V (DAJ; 1993) using the dmft/DMFT index (the single‐value sum of the decayed D(d), missing M(m), and filled F (f)(teeth). To access plaque accumulation, the observations after applying a plaque revelator were scaled into five classes (PlaqSearch, TePe®, Tepe D‐A‐CH GmbH, Hamburg, Germany): 0: no plaque; 1: isolated plaque islands; 2: a contiguous plaque line up to 1 mm thick at the gingival margin; 3: plaque expansion in the cervical tooth thirds; 4: plaque expansion to the middle third; and 5: plaque expansion to the coronal third.

Furthermore, the SCASS (Schiff, Dotson, Cohen, De WV, & Volpe, 1994) and the WBFS^20^ were used to determine the sensitivity of teeth to an air stimulus and to test personal pain experience. For standardized data collection, the SCASS was categorized according to the patient's reaction when applying a dental unit air syringe (pressure: 60 ± 5 psi, temperature: 20 ± 2°C, and application: 1–2 s at a distance of 1 cm from the tooth). The grading was 0 (no response), 1 (response but no removal request), 2 (removal request and patient movement away from the airflow), or 3 (removal request, leaning away from the stimulus and a feeling of pain). In addition to the examination of all MIH‐affected teeth, every unaffected tooth from the same dental group (e.g., all first molars) was also evaluated. During the same procedure, the WBFS score was found as a measurement for the subjective experience of pain. The patients were asked to match their intensity perception to a display showing a scale reaching from a smiling (score 0) to a crying (score 10) face (Wong & Baker, 1988).

The study participants and their legal guardians were interviewed regarding MIH‐/DMH‐dependent PwIoF. The Child Perception Questionnaire (Bekes, John, Schaller, & Hirsch, 2011) served as the basis for documenting the patients' overall nutritional restrictions. The children were queried in greater detail regarding whether they experienced problems or pain with chewing solid food or with eating and drinking hot, cold, or spicy items. In terms of validation, one negative association was sufficient to characterize the patient as a restricted eater.

The patients' data summarizing the severity of the MIH/DMH, according to Mathu‐Muju and Wright (2006), and the number of affected teeth were classified into three severity categories. The patients were rated according to their MIH/DMH severity (Ebel et al., 2018): Mildly affected teeth were scored as 0 “risk points” as they did not affect the QHI (Quigley & Hein, 1962) and, therefore, the caries risk (TDR); moderately affected teeth were rated with 2 points; and severely affected teeth received 3 points. The sum of risk points per person was calculated to determine the personal severity score, which combines the evaluated number and decay of affected teeth. Thereafter, the patients were classified within an LSC if they had a score of 0, within the MSC with a score of 2–4 points, or within the HSC with a score of >4 points.

The MIH‐TNI, as described by Steffen et al. (2017), was used to compare the impacts of different treatments. Patients with no hypomineralization were classified as MIH‐TNI 0; MIH‐affected patients without hypersensitivity or enamel defects were classified as MIH‐TNI 1; patients with MIH and without hypersensitivity but with enamel defect were classified as MIH‐TNI 2; patients with MIH and hypersensitivity but without enamel defect were classified as MIH‐TNI 3; and MIH‐affected patients with both hypersensitivity and enamel defects were classified as MIH‐TNI 4. A suitable therapy was chosen on the basis of each patient's MIH‐TNI status (Jälevik, 2010; Steffen et al., 2017).

### 2.7 Bias

The SCASS and WBFS values depend on the personal sensation of pain and can change with age or the examiner's proceedings. The QHI could be biased by the children's general teeth‐cleaning habits, such as additional brushing of teeth before the appointment and by the choice and application of the plaque‐disclosing agent. To prevent differences, the QHI was assessed with the same agent in every patient. The PwIoF could be affected by the children's general eating habits, including personal preferences for or against solid and spicy food. In terms of classifying therapy success, note that every child has a different personal pain tolerance and that an adaption of nutrition caused by persistent hypersensitivity is within the realm of possibility.

In this context, the possible occurrence of material application errors due to incorrect storage, use past the expiration date, or inaccurate usage and noncompliance with the manufacturer's recommended polymerization times could influence the therapeutic success. Further, young patients' personal behavior and compliance with therapy can interfere with the dentist's ranking (QHI, SCASS, WBFS, and PwIoF) and treatment methods (e.g., children who are being restless, fidgety, or extremely anxious can hinder the draining of the operating environment and complicate the treatment options and assessment of the indices and scores). Another limitation of the treatment can be inadequate levels of anesthesia, which my lead to difficulties in treatment due to the sensitivity of damaged teeth. In the case of an incomplete excavation, the restorative margins may not lie within the healthy tooth structure, which could cause painful side effects and, therefore, possible bias.

### 2.8 Statistical methods

In our follow‐up study, we examined the improvements to our quantitative measures with respect to different tooth groups (MIH) and patient groups (e.g., MSC) and various treatments. Progress was indicated by improved mean values of our measurements (using ANOVA) or by improved *Z* values (using the Wilcoxon test). The significance of the hypothesis using ANOVA was accepted if (a) the two confidence intervals did not overlap, (b) the mean of group A was not located within the confidence interval of group B, and (c) the *p* value was less than 5%. For subsequent Wilcoxon tests, significance for *p* values lower than 5% was accepted.

After acquiring information through examination or questioning, the data were dictated to a dental assistant, who transferred them to a database via an input mask (Microsoft Access 2016). The mask was used to build the platform for a lower error rate and allowed straightforward verification of the accuracy and completeness of the data. The survey results were transferred to and analyzed in Microsoft Excel 2016 in tabular form. The diagrams and graphics listed in this work were created with the same program.

## 3 RESULTS

### 3.1 Participants

In total, 78 children were included in the study, and the patients' genders were nearly equally distributed, with 37 female and 41 male patients. The mean age of the examined children was 8.5 years, ranging from 3 to 15 years of age, again with no significant difference concerning gender. Additionally, 15% (*n* = 12) of the patients were non‐Caucasian; 25.6% of the children (*n* = 20) could be assigned to a high social status, meaning that at least one parent had an academic degree; 61 children (78.2%) showed a normal dental caries risk; and 17 (21.8%) children were classified as high TDR. The mean dmft/DMFT was highest in the group of 6‐ to 9‐year‐old patients (2.83). Furthermore, 24 children (30.8%) could be assigned to the LSC, 26 (33.3%) to the MSC, and 28 (35.9%) to the HSC, reaching an almost equal sample size in the individual groups (Table 1).

**Table 1:** Summary of sample data (*n* = 78 patients)

	% (*n*)
Gender	
Male	52.6 (41)
Female	47.4 (37)
Age	
Ø	8.5 years
Range	3–15 years
Severity categories	
LSC	30.8 (24)
MSC	33.3 (26)
HSC	35.9 (28)
dmft/DMFT	
Mean dmft (3–5 years)	0.64 (11)
Mean dmft/DMFT (6–9 years)	2.83 (48)
Mean DMFT (10–15 years)	1.11 (19)
TDR	
High	21.8 (17)
Normal	78.2 (61)
Social status	
High	25.6 (20)
Normal	74.4 (58)

Abbreviations: HSC, high‐severity category; LSC, low‐severity category; MSC, medium‐severity category; TDR, tooth decay risk.

### 3.2 Main results

A total of 1,831 teeth were included in the study, comprising 865 (47.2%) deciduous and 966 (52.8%) permanent teeth. Among them, 219 (12%) teeth were MIH positive, of which 9.1% (*n* = 20) were deciduous and 90.9% (*n* = 199) were permanent. The differentiation of MIH teeth according to their severity showed that 105 teeth could be classified as MIH Severity Group I (48%), 66 teeth as Severity Group II (30.1%), and 48 teeth as Severity Group III (21.9%; Appendix A). The MIH‐TNI (Jälevik, 2010; Steffen et al., 2017) was used as a guideline to choose comparable therapeutic approaches for patients suffering from different consequences of the hypomineralization (Table 2). Most of the teeth (*n* = 107) were in the TNI 1 class, excluding hypersensitivity and posteruptive enamel breakdowns. Additionally, 52 of the TNI 1 teeth received only fluoride varnish as prophylaxis, another 50 teeth had fissure sealants, and five teeth were filled. The two teeth graded TNI 2 were restored with fillings. The TNI 3 group consisted of 30 teeth, among which 19 received fissure sealants, six were treated with fillings, and five had fluoride applied in varnish form. TNI 4 teeth suffering from hypersensitivity and enamel breakdown were mainly restored with fillings (*n* = 48) or stainless steel crowns (*n* = 26).

**Table 2:** Differentiation of customized therapy (tooth related) based on the Molar Incisor Hypomineralisation Treatment Need Index (Steffen et al., 2017)

	TNI 1	TNI 2	TNI 3	TNI 4
Total	107	2	30	80
Fluoride varnish	52	0	5	4
Fissure sealant	50	0	19	1
Filling	5	2	6	48
SSC	0	0	0	26
Extraction	0	0	0	1

Abbreviations: SSC, stainless steel crown; TNI, Treatment Need Index.

Table 3 shows the therapeutic approaches according to the severity classification of the 219 MIH teeth. Thereby, the MIH severity group was positively correlated with the chosen therapy strategy (chi‐squared test: *p* = .00) defined by the TNI (Jälevik, 2010; Steffen et al., 2017). The majority of teeth treated with fluoride varnish within a prophylaxis session (*n* = 61) were classified as MIH class I teeth (86.9%). Among the obtained teeth, 70 were treated with fissure sealants, whereas fillings were applied to 61 teeth, most of which were classified as MIH I or II. The 26 teeth that needed a stainless steel crown could be assigned to MIH Class III in most cases (80.8%). In addition, one tooth within the MIH III category was not worth retaining and had to be removed. As a consequence, this tooth was excluded from further evaluation, leaving a group size of 218 MIH‐positive teeth.

**Table 3:** Differentiation of customized therapy (tooth related) for MIH‐affected teeth (Mathu‐Muju & Wright, 2006, *n* = 219 teeth)

	All		MIH I		MIH II		MIH III	
	*n*	%	*n*	%	*n*	%	*n*	%
Total	219	100.0	105	48.0	66	30.1	48	21.9
Fluoride varnish	61	27.8	53	86.9	8	13.1	0	0.0
Fissure sealant	70	32.0	49	70.0	21	30.0	0	0.0
Filling	61	27.8	3	4.9	32	52.5	26	42.6
SSC	26	11.9	0	0.0	5	19.2	21	80.8
Extraction	1	0.5	0	0.0	0	0.0	1	100.0

Abbreviations: MIH, molar incisor hypomineralization; SSC, stainless steel crown.

Deeper analysis included 218 MIH‐positive teeth, which were differentiated by the allocated therapeutic approach and by the MIH severity before and after the treatment (Tables 4, 5, 6). Table 4 presents a significantly decreased QHI in all categories after treatment, with the exception of the prophylaxis (fluoride varnish). The more severe the MIH is, the better the QHI range after completion of the treatment, decreasing from QHI 2.56 to 2.11 in teeth treated with sealants, compared with a QHI from 4.19 to 2.54 in teeth prepared with stainless steel crowns. Furthermore, the QHI was significantly decreased after therapy in all MIH severity categories. The validation of the SCASS (Table 5) resulted in statistically consistent values before and after treatment if the teeth were treated with fluoride varnish (from 0.34 to 0.25) or fissure sealants (0.66 to 0.64). Teeth that were provided with fillings (SCASS from 1.87 to 1.56) or steel crowns (2.50 to 1.77) showed significantly decreased hypersensitivity. Similar results were obtained using the WBFS to assess the hypersensitivity (Table 6). In teeth treated with fluoride varnish and sealants, the WBFS decreased but showed no statistical significance, whereas teeth provided with fillings (WBFS 5.34 to 2.51) or crowns (WBFS 6.81 to 2.46) showed clear improvements. In addition, MIH II teeth also showed a decreased WBFS (4.38 to 2.18). Determination of the SCASS and WBFS scores before and after treatment revealed the biggest improvements in terms of hypersensitivity in the MIH III category (SCASS 2.32 to 1.70 and WBFS 6.60 to 3.11).

**Table 4:** Test for significant differences between the mean Quigley Hein Index before and after therapy (tooth related), including 95% CIs

	ANOVA						Wilcoxon
	*n*	Before		After			
Therapy	218	Mean	95% CI	Mean	95% CI	Difference	*p* value
Fluoride varnish	61	2.15	[1.91, 2.39]	2.05	[1.81, 2.29]	0.10	.40
Fissure sealant	70	2.56	[2.33, 2.78]	2.11	[1.89, 2.34]	0.45	.00
Filling	61	3.52	[3.28, 3.77]	2.23	[1.99, 2.47]	1.29	.00
SSC	26	4.19	[3.82, 4.56]	2.54	[2.17, 2.91]	1.65	.00

	*n*	Before		After			
MIH^a^	218	Mean	95% CI	Mean	95% CI	Difference	*p* value
MIH I	105	2.13	[1.96, 2.31]	1.94	[1.76, 2.12]	0.19	.03
MIH II	66	3.35	[3.13, 3.57]	2.30	[2.08, 2.53]	1.05	.00
MIH III	47	4.02	[3.76, 4.28]	2.53	[2.26, 2.80]	1.49	.00

Abbreviations: ANOVA, analysis of variance; CI, confidence interval; MIH, molar incisor hypomineralization; SSC, stainless steel crown.

a Mathu‐Muju and Wright (2006).

**Table 5:** Test for significant differences between the mean Schiff Cold Air Sensitivity Scale before and after therapy (tooth related), including 95% CIs

Therapy	ANOVA						Wilcoxon
	*n*	Before		After			
	218	Mean	95% CI	Mean	95% CI	Difference	*p* value
Fluoride varnish	61	0.34	[0.16, 0.53]	0.25	[0.04, 0.45]	0.09	.01
Fissure sealant	70	0.66	[0.48, 0.83]	0.64	[0.45, 0.84]	0.02	.74
Filling	61	1.87	[1.68, 2.05]	1.56	[1.35, 1.77]	0.31	.03
SSC	26	2.50	[2.22, 2.78]	1.77	[1.45, 2.09]	0.73	.01

	*n*	Before		After			
MIH^a^	218	Mean	95% CI	Mean	95% CI	Difference	*p* value
MIH I	105	0.29	[0.16, 0.41]	0.25	[0.10, 0.40]	0.04	.32
MIH II	66	1.62	[1.46, 1.78]	1.44	[1.25, 1.63]	0.18	.14
MIH III	47	2.32	[2.13, 2.51]	1.70	[1.48, 1.92]	0.62	.00

Abbreviations: ANOVA, analysis of variance; CI, confidence interval; MIH, molar incisor hypomineralization; SSC, stainless steel crown.

a Mathu‐Muju and Wright (2006).

**Table 6:** Test for significant differences between the mean Wong–Baker Faces Scale before and after therapy (tooth related), including 95% CIs

Therapy	ANOVA						Wilcoxon
	*n*	Before		After			
	218	Mean	95% CI	Mean	95% CI	Difference	*p* value
Fluoride varnish	61	1.16	[0.67, 1.66]	1.07	[0.57, 1.56]	0.09	.13
Fissure sealant	70	1.63	[1.16, 2.09]	1.24	[0.78, 1.71]	0.39	.02
Filling	61	5.34	[4.85, 5.84]	2.51	[2.01, 3.01]	2.83	.00
SSC	26	6.81	[6.05, 7.57]	2.46	[1.70, 3.22]	4.35	.00

MIH^a^	*n*	Before		After			
	218	Mean	95% CI	Mean	95% CI	Difference	*p* value
MIH I	105	0.85	[0.51, 1.19]	0.75	[0.40, 1.11]	0.10	.16
MIH II	66	4.38	[3.95, 4.81]	2.18	[1.74, 2.63]	2.20	.00
MIH III	47	6.60	[6.09, 7.10]	3.11	[2.58, 3.63]	3.49	.00

Abbreviations: ANOVA, analysis of variance; CI, confidence interval; MIH, molar incisor hypomineralization; SSC, stainless steel crown.

a Mathu‐Muju and Wright (2006).

Table 7 provides an overview of the QHI changes per person before and after the application of each therapeutic approach. Children in the LSCs did not benefit from the medical care (LSC: from 1.12 to 1.16), whereas the mean plaque accumulation of patients classified within the MSC or HSC showed improvements (MSC: from 2.15 to 1.96; HSC: from 2.67 to 2.20). Therefore, the reduction of QHI in the HSC group was statistically significant. A change in the mean QHI of greater or less than 0.5 indicated a declined or improved condition, and 75% of the children in the LSC showed constant plaque accumulation before and after care. Although a similar constant pattern was assessed for patients from the MSC (73.1%), half of the patients in the HSC showed improvement after therapy (Appendix F).

**Table 7:** Comparison of mean Quigley Hein Index (patient related) before and after therapy concerning severity categories (Ebel et al., 2018)

Severity category	ANOVA						Wilcoxon
	*n*	Before		After			
	78	Mean	95% CI	Mean	95% CI	Difference	*p* value
Low	24	1.12	[0.86, 1.37]	1.16	[0.89, 1.44]	−0.04	.81
Medium	26	2.15	[1.90, 2.39]	1.96	[1.70, 2.23]	0.19	.04
High	28	2.67	[2.43, 2.91]	2.20	[1.94, 2.45]	0.47	.00

Abbreviations: ANOVA, analysis of variance; CI, confidence interval.

Table 8 characterizes the MSC and HSC according to their problems with food intake before and after specialized treatment. In both groups, therapy resulted in a significant decrease in PwIoF. Before treatment, 12 patients from the MSC and 21 patients from the HSC suffered from general PwIoF, compared with seven MSC patients and 14 HSC patients after medical care. Differentiation of the problems shows significant improvement for children in the HSC and for patients who felt restricted in terms of chewing hot or cold (*p* = .09) or spicy food (*p* = .02) but not for children who had problems with eating solid food. Primarily in the MSC, therapy reduced problems with eating hot or cold food from 46.2% to 26.9%.

**Table 8:** Ratios for different severity categories (patient related) concerning PwIoF before and after therapy

	Wilcoxon					
	MSC			HSC		
	33.3 (26)			35.9 (28)		
	Before	After		Before	After	
Total *n* = 78	% (*n*)	% (*n*)	*p* value	% (*n*)	% (*n*)	*p* value
PwIoF “yes”	46.2 (12)	26.9 (7)	0.00	75.0 (21)	50.0 (14)	.01
PwIoF hot or cold food “yes”	46.2 (12)	26.9 (7)	0.03	75.0 (21)	39.3 (11)	.09
PwIoF solid food “yes”	11.5 (3)	3.9 (1)	0.05	57.1 (16)	10.7 (3)	.20
PwIoF spicy food “yes”	11.5 (3)	2.6 (2)	0.16	39.3 (11)	0.0 (0)	.02

*Note.* There were no PwIoF in the low‐severity category.

Abbreviations: HSC, high‐severity category; MSC, medium‐severity category; PwIoF, problems with the intake of food.

Patients in the LSC did not have any problems with food intake (Appendix E). Although PwIoF status did not change in 73.1% of children from the MSC, participants grouped in the HSC benefited from therapy in 67.9% of the cases.

Additionally, the number of teeth was grouped by therapeutic success in terms of decreasing QHI, SCASS, and WBFS scores (Appendices B–D). The specific treatment ranged from prophylaxis to the insertion of stainless steel crowns. Teeth restored with crowns showed a predominant improvement in all scales (QHI: 80.8%, SCASS: 61.5%, and WBFS: 88.5% of teeth), whereas the percentage of teeth with fillings mainly showed improvements in a lower QHI (80.3%) and in a lower WBFS (78.7%). The teeth that were treated with milder therapy (fluoride varnish and fissure sealants) mostly maintained their scores on the QHI, SCASS, and WBFS, including a range from 47.1% to 90.2% of teeth with constant values. Teeth ranked as MIH I showed mainly constant values of QHI (54.3%), SCASS (83.8%), and WBFS (89.5%) before and after treatment, whereas teeth placed in MIH Classes II and III were mostly characterized by improved values. Patients with MIH III teeth benefited the most from the treatment: 83% of the MIH III teeth showed less plaque accumulation (QHI), 48.9% had a lower SCASS score, and 80.9% had a lower rating on the WBFS. Children grouped into the LSC mainly stayed at the same level in terms of plaque accumulation (QHI: 75%) and PwIoF (100%). The same trend was prominent among the children grouped into the MSC. Most of the patients showed neither an improvement nor a decline in QHI (73.1%) or PwIoF (73.1%). The 28 patients from the HSC benefited the most from the specialized treatment, characterized by an improved QHI (50%) and PwIoF (67.9%).

## 4 DISCUSSION

The present study demonstrates significant correlations between the severity of MIH, the therapeutic approach applied, the presence of hypersensitivity, and changes in food intake. MIH and DMH show several side effects, such as the exposure of dentin due to enamel breakdown, which might lead to increased hypersensitivity and decreased oral hygiene. Furthermore, this results in a higher risk of developing dentin caries. Moreover, patients can suffer from problems during eating or drinking of hot, cold, spicy, or particularly solid food (Ebel et al., 2018). Specialized medical care is essential for improving MIH‐affected patients' situations. Therefore, dentists worldwide are challenged by the differentiation of the MIH‐affected area and must prevent further breakdown and secondary caries as well as choose the optimal therapeutic approach, material, and time point for children's treatment (Kohlboeck, Heitmueller, Neumann, et al., 2013; Krishnan & Ramesh, 2014; Mathu‐Muju & Wright, 2006; Newman, 1986; Walsh, 2006). As the etiology of MIH presently remains unclear (Silva, Scurrah, Craig, Manton, & Kilpatrick, 2016; Taylor, 2017), only early diagnosis and treatment at the start of dentition can ensure the best possible therapy. Unfavorably, clinicians' seem to have a broad disparity of opinions on the best treatment options for MIH‐affected teeth (Kopperud, Pedersen, & Espelid, 2017). The best option for these children is to prevent posteruptive risks, including by monitoring for cariogenic nutrition, balanced fluoride levels, and pH values in the oral region, together with sufficient oral hygiene and regular monitoring by a dentist. The high incidence of MIH‐affected patients can be explained by the location and specialization of the practice. It is the only specialized pediatric practice in a densely populated area of North Rhine‐Westphalia, with a total recruitment area of 100,000 inhabitants that treats children requiring general anesthesia or major rehabilitation. Taking the patient pool as an advantage for this study, the severity categories were based on equal participant numbers, including patients suffering from the highest MIH score.

The MIH‐TNI provides standardized guidelines for choosing treatment options according to MIH status, as the index was designed for this purpose (Jälevik, 2010; Steffen et al., 2017). This study evaluated the recommended treatment options according to the improvements after therapy.

In mild cases, fluoride varnish was the treatment of choice and resulted in enamel strengthening, which can contribute to the prevention of caries. All patients treated with this approach were recommended for therapy of the MIH‐affected teeth. However, some parents or legal guardians rejected a specific therapy for MIH teeth, especially in cases without hypersensitivity or general pain or if the recommended therapy would have included anesthesia. These children with teeth ranked in the TNI 3 (*n* = 5) and 4 (*n* = 4) groups were monitored and treated with prophylaxis. The analysis of plaque accumulation and hypersensitivity before and after treatment (OHI: 2.15 to 2.05, SCASS: 0.34 to 0.25, and WBFS: 1.16 to 1.07) confirmed the absence of suffering.

Sealants were used to reduce plaque accumulation on fissures and to improve sensitivity within exposed areas through adhesive mounting. Defect‐oriented restorations for maintaining tooth vitality were performed in cases of exposed dentin and/or the development of caries. Around one third of the teeth in the MIH I group and two thirds of the teeth in the MIH II group were treated with sealants. All parameters decreased after treatment. Hypersensitivity was higher in this particular group, as represented in the upper values of the SCASS and WBFS, compared with teeth to which fluoride varnish was applied. The higher plaque accumulation in this group could also be explained by the increased hypersensitivity and the observation that fissure sealants often need to be restored, which further promotes lasting sensitivity.

The teeth treated with fillings were mainly grouped into MIH II (*n* = 32) and III (*n* = 26), with an MIH‐TNI of 4 (*n* = 48). The high QHI scores before treatment support the classification because the tooth surfaces were generally rough but improved with treatment (3.52 to 2.23). The recommended treatment showed large improvements in this group, as the subjective feeling of pain (WBFS 5.34 to 2.51) and the SCASS score (1.87 to 1.56) both significantly decreased. Nevertheless, fillings often result in posttreatment side effects, such as prolonged feelings of pain or sensitivity due to drilling. These effects can be the reason for an extended recovery time, which can result in a longer postoperative time period (e.g., 6 months) until the follow‐up examination.

Stainless steel crowns were used if more than two thirds of the tooth surface was damaged, if the patient's hypersensitivity would not allow for the application of a filling, or if the filling did not produce any improvement in the patient's status. Mainly MIH III teeth classified as TNI 4 were provided with crowns. The application of the crown immediately evened out rough and porous surfaces, explaining the striking decrease in the QHI score from 4.19 to 2.54 in this study. Furthermore, the SCASS (2.5 to 1.77) and WBFS (6.81 to 2.46) scores showed significant decreases, supporting this choice of crown application for severe cases. The pain that these children experienced could mainly be reduced by applying crowns.

Recommendations for extraction and optimized teeth position were obtained from Jälevik and Möller, who suggested extraction if the pulpitis was irreversibly damaged, the defect was too subgingival, or treatment was not possible under inhalation anesthesia (Jälevik, 2010; Steffen et al., 2017). In this study, only one of 219 teeth fulfilled these criteria and had to be removed.

Note that pain perception and restrictions are most likely to adapt as the children age, as this study lasted for 1 year. The estimation of felt pain (WBFS) and the reaction to an air stimulus (SCASS) are not free of personal bias, especially if the children experienced several treatment sessions including drilling or anesthesia and became accustomed to the procedure.

This study focused on the comparability between different severely damaged teeth and patient suffering. Therefore, participants were nearly equally distributed into the three severity categories, from LSC to HSC. Patients grouped into the LSC and MSC included only a few mild to moderate cases of MIH teeth per patient, with a relatively small impact on overall oral hygiene. Consequently, patients from the LSC and MSC groups did not show great changes in the QHI score, whereas children from the HSC were characterized by severe cases of MIH teeth or had several damaged teeth. In comparison, these severely hypomineralized teeth had a greater impact on overall oral hygiene. Therefore, these HSC patients showed the greatest improvement after therapy, with a mean QHI score decrease from 2.67 to 2.20.

A similar picture can be seen in terms of nutritional restrictions. Patients from the LSC were not but children from the MSC and HSC were challenged with PwIoF. Thus, the HSC showed the best improvements following the application of specific therapeutic approaches. Treatment could decrease the hypersensitivity of the teeth and thermal stimuli, and spiciness and chewing became less painful for the children.

The results of this study unveiled how patients suffering heavily from MIH‐affected teeth in terms of plaque accumulation, hypersensitivity, and nutritional restrictions benefited the most from specific therapeutic approaches. The improvements in the severe cases further support the use of the Treatment Need Index (Jälevik, 2010; Steffen et al., 2017) as a guideline for therapy. A more standardized strategy by which to treat MIH could provide significant benefits to all children suffering from this enamel disorder and offer support to dentists worldwide in terms of choosing a form of therapy (Kopperud et al., 2017). Although this study could benefit many children, severely damaged teeth are not completely symptom free after the treatment of choice and must be monitored over the long term. This finding shows, once more, that children suffering from MIH require additional care and persistent therapy from qualified dentists.

## 5 CONCLUSION

This study confirms the hypothesis that patients benefit from therapy for MIH‐damaged teeth and that treatment can reduce hypersensitivity and improve the ability to ensure oral hygiene. Additionally, the number of dietary restrictions decreased after therapy. All the examined effects were significant for individual affected teeth but rather low throughout the overall dentition. These results imply that more severely damaged teeth are in greater need of invasive therapy than mildly damaged ones. These results underline the importance of treating MIH‐damaged teeth to help relieve children's suffering and improve their quality of life.

## AUTHOR CONTRIBUTIONS

J. F., K. B., C. H., and M. E. conceived the study's premise. J. F. performed the clinical testing, led the data collection, and wrote the manuscript. C. K. analyzed the data, completed the statistical analysis, and wrote the statistical portion of the manuscript. K. B., C. H., C. K., and M. E. interpreted the results and critically reviewed the manuscript.

## CONFLICT OF INTEREST

The authors do not have any financial interest in the companies whose materials are included in this article.

## CLINICAL SIGNIFICANCE

To minimize hypersensibility, MIH‐affected teeth require selective therapy, depending on the severity of the damage. This is the only way to improve children's oral hygiene and to minimize limitations to food intake. Prior categorization of damaged teeth using the MHI‐TNI method simplifies the search for a suitable therapy.

## APPENDIX A

### SUMMARY TABLE OF TOOTH DATA IN 78 PATIENTS WITH MIH (MATHU‐MUJU & WRIGHT, 2006, *N*=1,831 TEETH)

**Table:**

(n = 1831 teeth)	
	% (*n*)
Dentition	
Primary teeth	47.2 (865)
Permanent teeth	52.8 (966)
MIH affected	12.0 (219)
Primary teeth	9.1 (20)
Permanent teeth	90.9 (199)
MIH I	48.0 (105)
MIH II	30.1 (66)
MIH III	21.9 (48)

Abbreviation: MIH, molar incisor hypomineralization.

## APPENDIX B

### CHANGE IN THE MEAN QUIGLEY HEIN INDEX BEFORE AND AFTER THERAPY (TOOTH RELATED)

**Table:**

Therapy	*n*	Declined	Constant	Improved
	218	% (*n*)	% (*n*)	% (*n*)
Fluoride varnish	61	18.0 (11)	54.1 (33)	27.9 (17)
Fissure sealant	70	10.0 (7)	47.1 (33)	42.9 (30)
Filling	61	4.9 (3)	14.8 (9)	80.3 (49)
SSC	26	11.5 (3)	7.7 (2)	80.8 (21)

MIH^a^	*n*	Declined	Constant	Improved
	218	% (*n*)	% (*n*)	% (*n*)
MIH I	105	15.2 (16)	54.3 (57)	30.5 (32)
MIH II	66	7.6 (5)	22.7 (15)	69.7 (46)
MIH III	47	6.4 (3)	10.6 (5)	83.0 (39)

a Mathu‐Muju and Wright (2006).

*Note.* We defined declined/improved as a deviation greater/lower than 0.5 mean QHI.

Abbreviations: MIH, molar incisor hypomineralization; SSC, stainless steel crown.

## APPENDIX C

### CHANGE IN THE MEAN SCHIFF COLD AIR SENSITIVITY SCALE BEFORE AND AFTER THERAPY(TOOTH RELATED)

**Table:**

Therapy	*n*	Declined	Constant	Improved
	218	% (*n*)	% (*n*)	% (*n*)
Fluoride varnish	61	0.0 (0)	90.2 (55)	9.8 (6)
Fissure sealant	70	10.0 (7)	75.7 (53)	14.3 (10)
Filling	61	16.4 (10)	47.5 (29)	36.1 (22)
SSC	26	23.1 (6)	15.4 (4)	61.5 (16)

	*n*	Declined	Constant	Improved
MIH^a^	218	% (*n*)	% (*n*)	% (*n*)
MIH I	105	4.8 (5)	83.8 (88)	11.4 (12)
MIH II	66	16.7 (11)	54.5 (36)	28.8 (19)
MIH III	47	14.9 (7)	36.2 (17)	48.9 (23)

a Mathu‐Muju and Wright (2006).

*Note.* We defined declined/improved as a deviation greater/lower than 1.

Abbreviations: MIH, molar incisor hypomineralization; SSC, stainless steel crown.

## APPENDIX D

### CHANGE IN THE MEAN WONG–BAKER FACES SCALE BEFORE AND AFTER THERAPY (TOOTH RELATED)

**Table:**

Therapy	*n*	Declined	Constant	Improved
	218	% (*n*)	% (*n*)	% (*n*)
Fluoride varnish	61	1.6 (1)	91.8 (56)	6.6 (4)
Fissure sealant	70	5.7 (4)	64.3 (45)	30.0 (21)
Filling	61	3.3 (2)	18.0 (11)	78.7 (48)
SSC	26	7.7 (2)	3.8 (1)	88.5 (23)

MIH^a^	*n*	Declined	Constant	Improved
	218	% (*n*)	% (*n*)	% (*n*)
MIH I	105	1.9 (2)	89.5 (94)	8.6 (9)
MIH II	66	4.6 (3)	21.2 (14)	74.2 (49)
MIH III	47	8.5 (4)	10.6 (5)	80.9 (38)

a Mathu‐Muju and Wright (2006).

*Note.* We defined declined/improved as a deviation greater/lower than 1.

Abbreviations: MIH, molar incisor hypomineralization; SSC, stainless steel crown.

## APPENDIX E

### CHANGE IN THE MEAN NUMBER OF PWLOF BEFORE AND AFTER THERAPY (PATIENT RELATED)

**Table:**

PwIoF Ø						
		Before	After	Declined	Constant	Improved
	*n*	% (*n*)	% (*n*)	% (*n*)	% (*n*)	% (*n*)
Total	78	42.3 (33)	26.9 (21)	6.4 (5)	60.3 (47)	33.3 (26)
LSC	24	0.0 (0)	0.0 (0)	0.0 (0)	100.0 (24)	0.0 (0)
MSC	26	46.2 (12)	26.9 (7)	0.0 (0)	73.1 (19)	26.9 (7)
HSC	28	75.0 (21)	50.0 (14)	17.9 (5)	14.3 (4)	67.9 (19)

Abbreviations: HSC, high‐severity category; LSC, low‐severity category; MSC, medium‐severity category; PwIoF, problems with the intake of food.

## APPENDIX F

### CHANGE IN THE MEAN QUIGLEY HEIN INDEX BEFORE AND AFTER THERAPY (PATIENT RELATED)

**Table:**

Severity category	*n*	Declined	Constant	Improved
	78	% (*n*)	% (*n*)	% (*n*)
Low	24	8.3 (2)	75.0 (18)	16.7 (4)
Medium	26	7.7 (2)	73.1 (19)	19.2 (5)
High	28	7.1 (2)	42.9 (12)	50.0 (14)

*Note.* We defined declined/improved as a deviation greater/lower than 0.5 mean Quigley Hein Index.
